# Supplementary material for: Structural insights into peptidoglycan hydrolysis by the FtsEX system in Escherichia coli during cell division
Source: eLife. 2026 Oct 28;13:RP94336. doi: 10.7554/eLife.94336 (PMC13716388; doi:10.7554/eLife.94336)
Supplement: Supplementary file 2 [file elife-94336-supp1.docx]

Table A. Oligonucleotides used in this study.

| Primer | Sequence (5’-3’) | Template |
| --- | --- | --- |
| **For construction of His_6_- FtsEX** | | |
| *Eco*EX-WT F | accaccaccatatgattcgctttgaacat | Genomic DNA of *E. coli K12 Str MG1655* |
| *Eco*EX-WT R | tgctcgacaagcttttattcaggcgtaaag |  |
| *Sp*EX-WT F | caccaccaccatatgtcaattattgaaat | Genomic DNA of *Streptococcus pneumoniae str. D39* |
| *SP*EX-WT R | ctcgacaagcttctaaatcttcaagaatcggc |  |
| *Pae*EX-WT F | ccaccaccaccatatgatccgcttcgagcagg | Genomic DNA of *Pseudomonas aeruginosa* *ATCC 47085D* |
| *Pae*EX-WT R | gctcgacaagctttcaagtgatcccggcggcg |  |
| *Bs*EX-WT F | ccaccaccatatgatagagatgaagga | Genomic DNA of *Bacillus_subtilis_subsp_subtilis_ATCC_23857*, |
| *Bs*EX-WT R | cgacaagcttttatactcgcagaaacttg |  |
| **For construction of His_6_-SUMO-EnvC** | | |
| EnvC-WT-F | tggtggatccgatgagcgtgaccaactc | Genomic DNA of *E. coli K12 Str MG1655* |
| EnvC-WT-R | tgctcgagtttatcttcccaaccacggct |  |
| **For construction of His_6_-SUMO-AmiB** | | |
| AmiB-WT-F | ggtggatccgcgacgctctctgatattc | Genomic DNA of *E. coli K12 Str MG1655* |
| AmiB-WT-R | gtgctcgagttagtttggcagcgtgcga |  |
| **For FtsE mutation** | | |
| *Eco*FtsE-E163Q-F | ggcggaccaaccgactggtaacctggacg | Vector-*Eco*FtsEX-WT |
| *Eco*FtsE-E163Q-R | cagtcggttggtccgccagcagtaccgcg |  |

**Table B. Plasmids used in this study.**

| **Plasmids** | **Promoter** | **Repressor** | **Fusion tags** | **Genes for overexpressed** | **Antibiotic resistance** | **Source** |
| --- | --- | --- | --- | --- | --- | --- |
| pTD68 | T7 | lac repressor | His6 | *EcoFtsEX, EcoFtsE^E163Q^X*  *PaeFtsEX*  *SpFtsEX*  *BsFtsEX* | Ampicillin | (1) |
| pET28-Sumo | T7 | lac repressor | His6-Sumo | *EnvC, AmiB* | Kanamycin | (2) |

Table C. Statistics of the cryo-EM structures presented in this study.

| **Cryo-EM data collection and processing** | **FtsEX/ATPγS** | **FtsE^E163Q^X/EnvC/ATP** |
| --- | --- | --- |
| Voltage(kV) | 300 | 300 |
| Electron dose (e^-^/Å^2^) | 35 | 39 |
| Physical pixel (Å) | 1.06 | 1.06 |
| Number of movies | 3084 | 6054 |
| Number of particles for final map | 116,651 | 182,732 |
| Resolution (Å) | 3.7 | 3.4 |
| Map B-factor (Å^2^) | -137 | -129 |
| **Model refinement** |  | |
| Number of protein residues | 1021 | 1127 |
| Number of chains | 4 | 5 |
| Number of atoms | 7752 | 8555 |
| Ligands | 2 ATPγS | 2 ATP |
| **Geometric parameters (r.m.s.d.）** |  | |
| Bond length (Å) | 0.004 | 0.005 |
| Bond angle (º) | 1.096 | 1.178 |
| **Ramachandran statistics** |  | |
| Residues favoured (%) | 94.65 | 91.94 |
| Residues allowed (%) | 5.05 | 7.61 |
| Residues disallowed (%) | 0.30 | 0.45 |
| Rotamer outliers (%) | 0.13 | 0.12 |
| **MolProbity Score** | 1.69 | 1.79 |

**References**

1.	T. Uehara, K. R. Parzych, T. Dinh, T. G. Bernhardt, Daughter cell separation is controlled by cytokinetic ring-activated cell wall hydrolysis. *EMBO J* **29**, 1412-1422 (2010).

2.	Y. I. Kim, D. J. Vinyard, G. M. Ananyev, G. C. Dismukes, S. S. Golden, Oxidized quinones signal onset of darkness directly to the cyanobacterial circadian oscillator. *Proc Natl Acad Sci U S A* **109**, 17765-17769 (2012).
