# Supplementary material for: Structural insights into peptidoglycan hydrolysis by the FtsEX system in Escherichia coli during cell division
Source: eLife. 2026 Oct 28;13:RP94336. doi: 10.7554/eLife.94336 (PMC13716388; doi:10.7554/eLife.94336)
Supplement: Supplementary file 3 [file elife-94336-fig1-data1.zip › Figure 1-source data 1.pdf]

Figure 1A

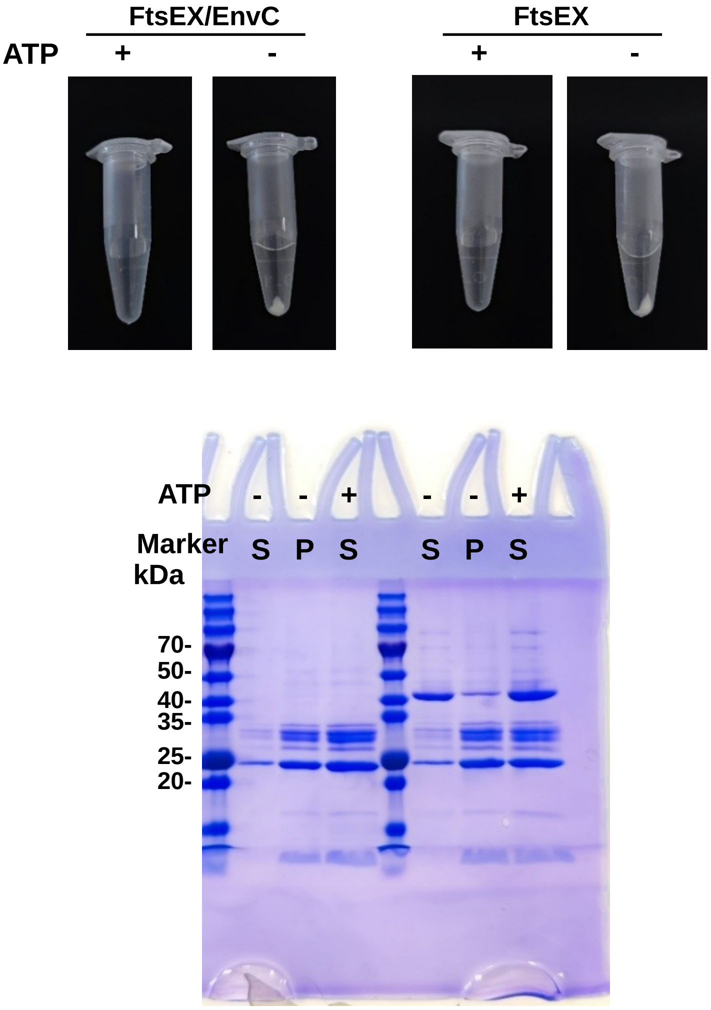

Figure1C

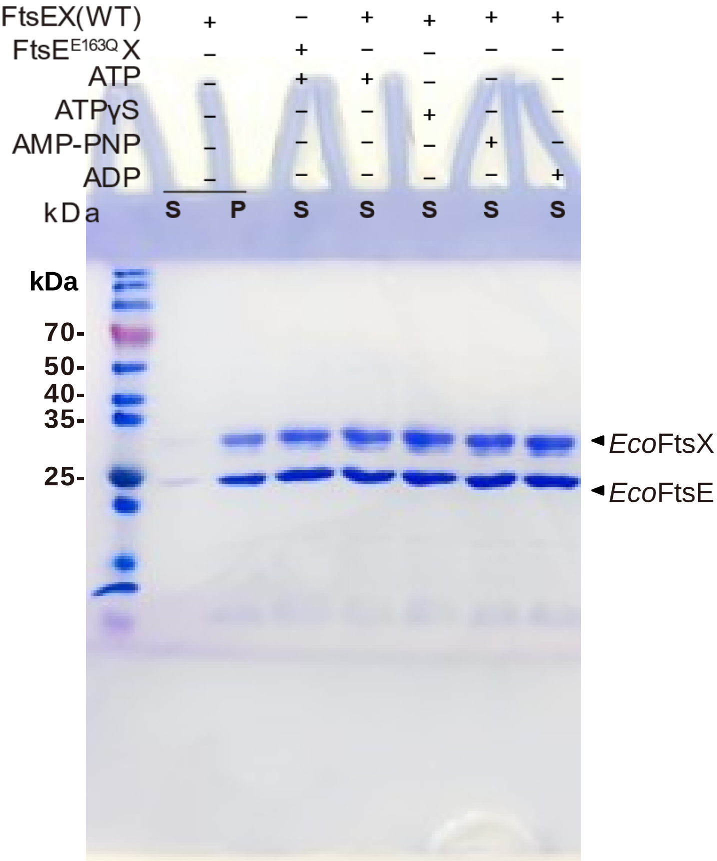

FtsEX(WT) + − + + + +
FtsE^{E163Q}X − + − − − −
ATP − + + − − −
ATPγS − − − + − −
AMP-PNP − − − − + −
ADP − − − − − +
kDa S P S S S S S
kDa
70-
50-
40-
35-
25-
EcoFtsX
EcoFtsE

Figure 1E

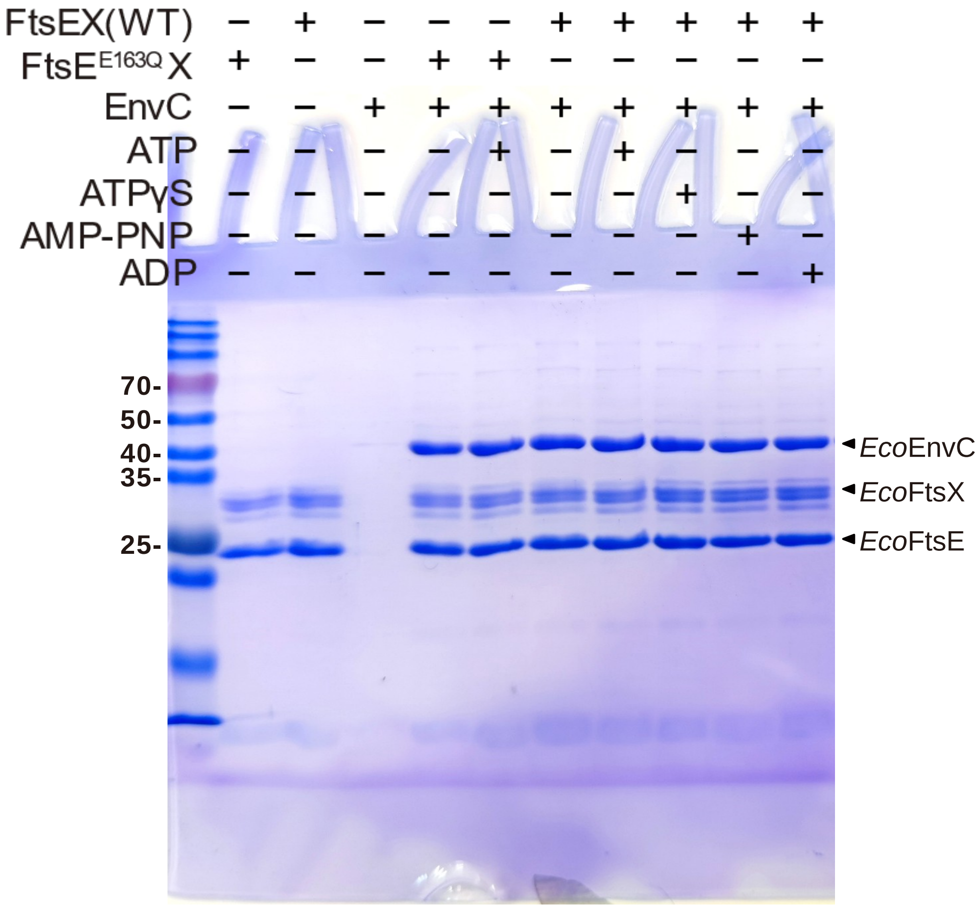

FtsEX(WT) − + − − − + + + + +
FtsE^{E163Q}X + − − + + − − − − −
EnvC − − + + + + + + + +
ATP − − − − + − + − − −
ATPγS − − − − − − − + − −
AMP-PNP − − − − − − − − + −
ADP − − − − − − − − − +
70-
50-
40-
35-
25-
*Eco*EnvC
*Eco*FtsX
*Eco*FtsE
