# Supplementary material for: Structural insights into peptidoglycan hydrolysis by the FtsEX system in Escherichia coli during cell division
Source: eLife. 2026 Oct 28;13:RP94336. doi: 10.7554/eLife.94336 (PMC13716388; doi:10.7554/eLife.94336)
Supplement: Supplementary file 5 [file elife-94336-fig1-figsupp1-data1.zip › Figure 1-figure supplement 1-source data 1.pdf]

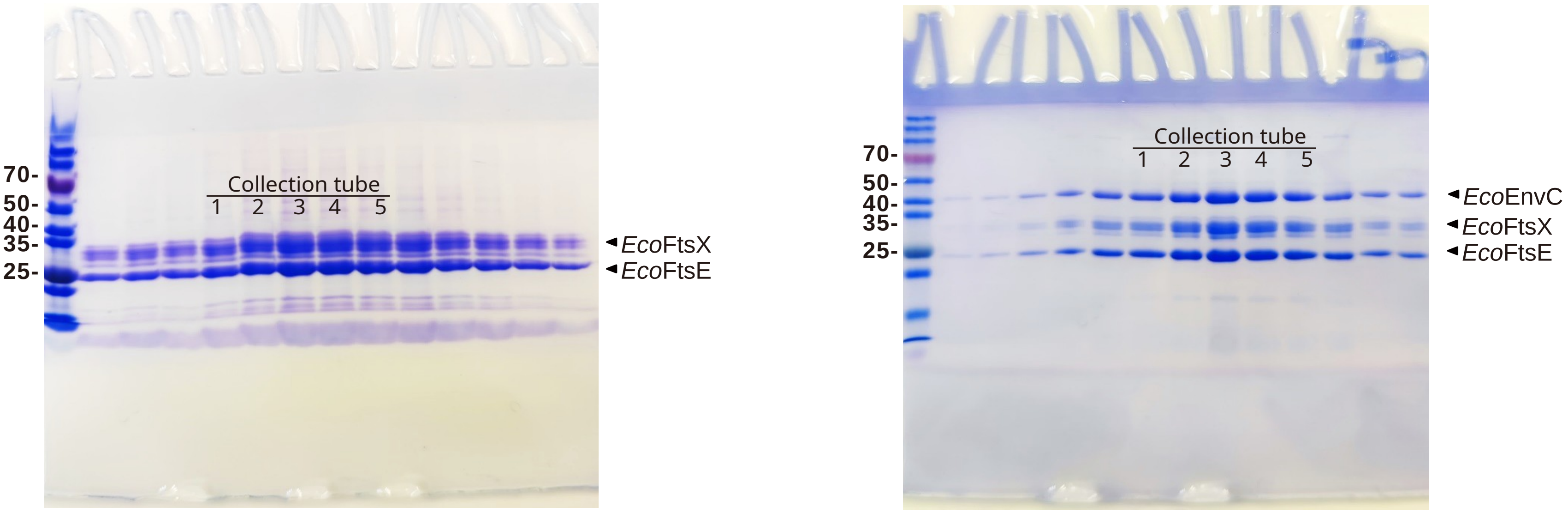
70-
50-
40-
35-
25-
Collection tube
1 2 3 4 5
◄EcoFtsX
◄EcoFtsE
70-
50-
40-
35-
25-
Collection tube
1 2 3 4 5
◄EcoEnvC
◄EcoFtsX
◄EcoFtsE

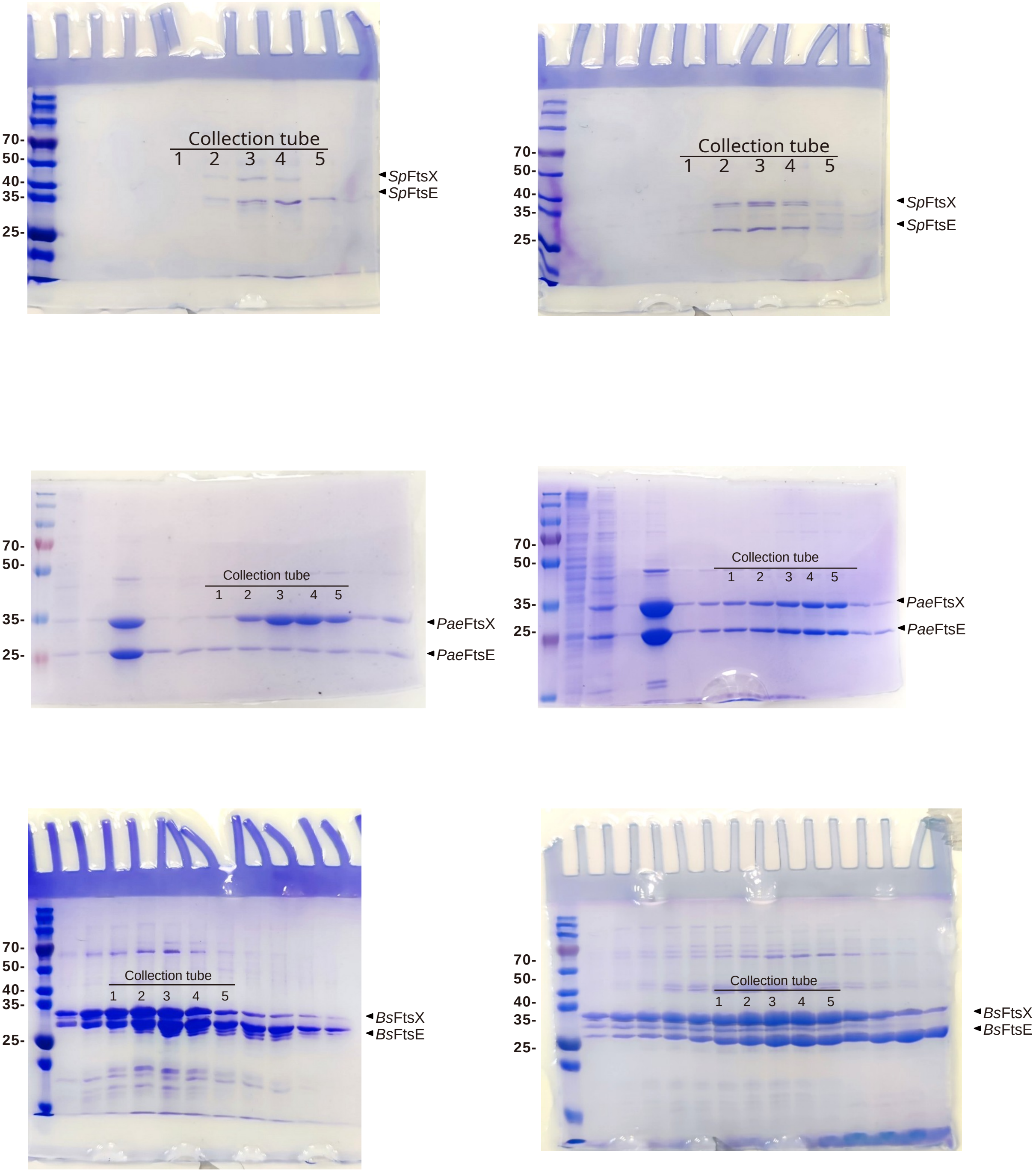

Collection tube
1 2 3 4 5
70-
50-
40-
35-
25-
SpFtsX
SpFtsE
Collection tube
1 2 3 4 5
70-
50-
40-
35-
25-
SpFtsX
SpFtsE
Collection tube
1 2 3 4 5
70-
50-
35-
25-
PaeFtsX
PaeFtsE
Collection tube
1 2 3 4 5
70-
50-
35-
25-
PaeFtsX
PaeFtsE
Collection tube
1 2 3 4 5
70-
50-
40-
35-
25-
BsFtsX
BsFtsE
Collection tube
1 2 3 4 5
70-
50-
40-
35-
25-
BsFtsX
BsFtsE
