# Supplementary material for: Structural insights into peptidoglycan hydrolysis by the FtsEX system in Escherichia coli during cell division
Source: eLife. 2026 Oct 28;13:RP94336. doi: 10.7554/eLife.94336 (PMC13716388; doi:10.7554/eLife.94336)
Supplement: Supplementary file 7 [file elife-94336-fig5-data1.zip › Figure 5-source data 1.pdf]

Figure 5A

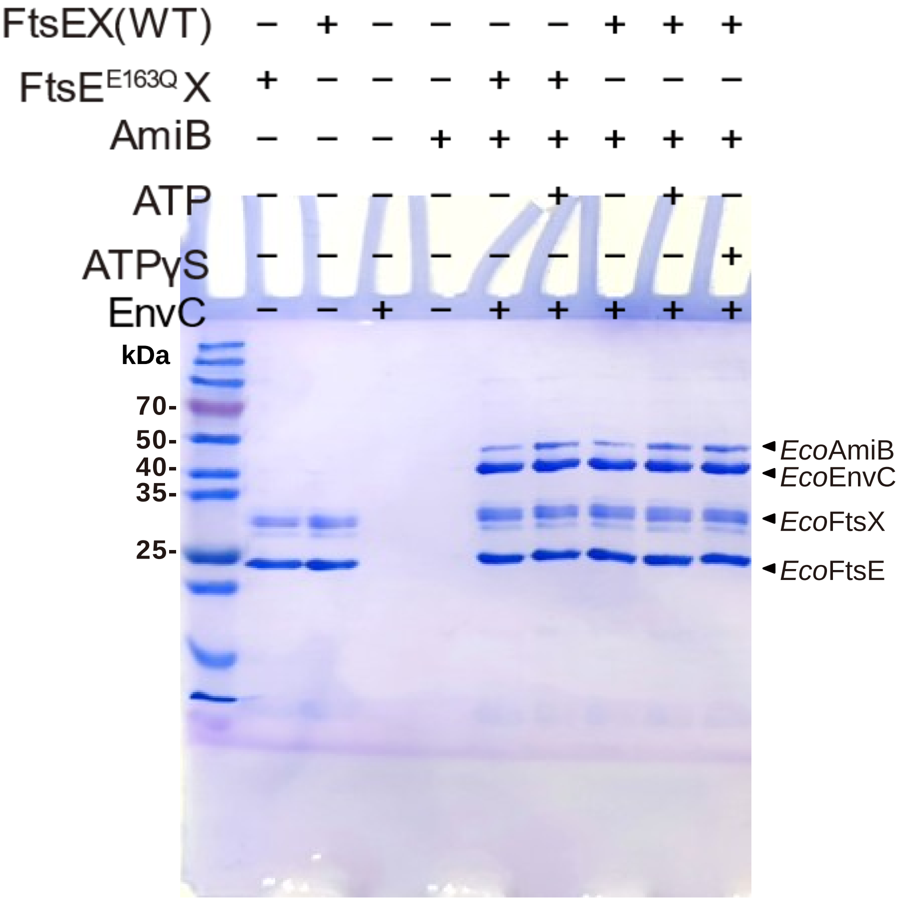

FtsEX(WT) − + − − − − + + +
FtsE^{E163Q}X + − − − + + − − −
AmiB − − − + + + + + +
ATP − − − − − + − + −
ATPγS − − − − − − − − +
EnvC − − + − + + + + +
kDa
70-
50-
40-
35-
25-
*Eco*AmiB
*Eco*EnvC
*Eco*FtsX
*Eco*FtsE

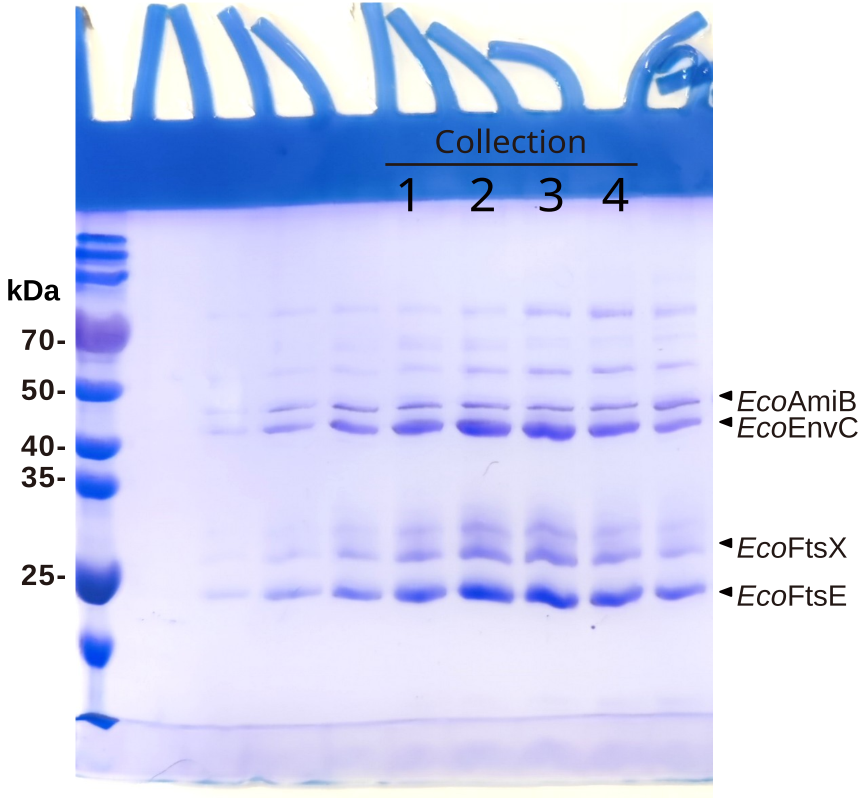
Collection
1
2
3
4
kDa
70-
50-
40-
35-
25-
*Eco*AmiB
*Eco*EnvC
*Eco*FtsX
*Eco*FtsE
