# Supplementary material for: Structural insights into peptidoglycan hydrolysis by the FtsEX system in Escherichia coli during cell division
Source: eLife. 2026 Oct 28;13:RP94336. doi: 10.7554/eLife.94336 (PMC13716388; doi:10.7554/eLife.94336)

Figure S9C

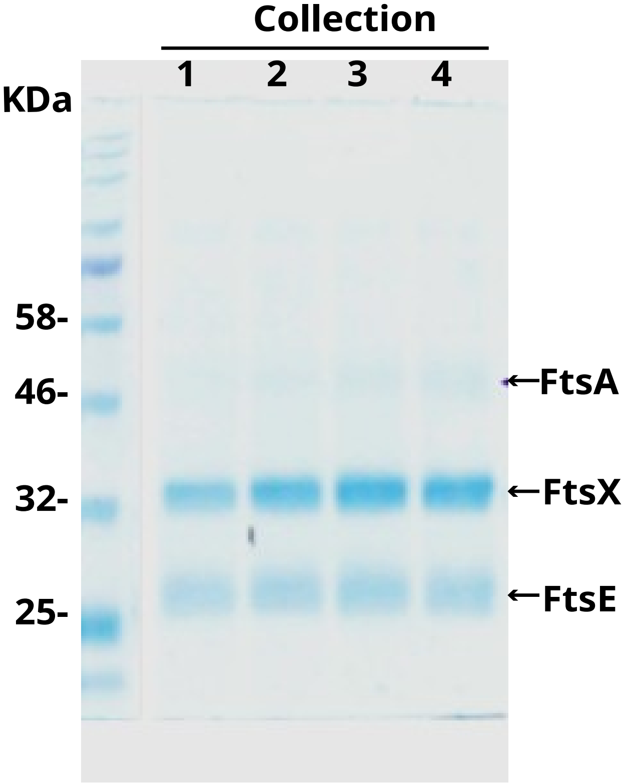

Supplement: Supplementary file 9 [file elife-94336-fig6-figsupp1-data1.zip › Figure 6-figure supplement 1-source data 1.pdf]
