# Supplementary material for: Structural insights into peptidoglycan hydrolysis by the FtsEX system in Escherichia coli during cell division
Source: eLife. 2026 Oct 28;13:RP94336. doi: 10.7554/eLife.94336 (PMC13716388; doi:10.7554/eLife.94336)
Supplement: Peer review history

# Editor report: eLife Assessment

**DOI:** 10.7554/eLife.94336.3.sa0

**Authors:** Amy H Andreotti

**Affiliations:** Iowa State University United States

**Keywords:** Solid, Useful

This is a potentially **useful** study that provides **solid**, yet confirmatory structural findings about the complex (FtsEX) that controls peptidoglycan remodeling during bacterial cell division. The authors capitalize on the fact that ATP binding stabilizes the FtsEX complex, allowing structural characterization for this system. A model is then developed to explain ATP regulation, but there is a gap between the model presented here and in vivo data reported previously.

# Referee report: Reviewer #1 (Public Review)

**DOI:** 10.7554/eLife.94336.3.sa1

Summary:

In this paper, Li and colleagues overcome solubility problems to determine the structure of FtsEX bound to EnvC from *E. coli*.

Strengths:

The structural work is well done, and the work is consistent with previous work on the structure of this complex from P. aerugionsa.

Weaknesses:

The model does not take into account all the information that the authors obtained, as well as known in vivo data.

The work lacks a clear comparison to the Pseudomonas structure highlighting new information that was obtained so that it is readily available to the reader.

The authors set out to obtain the structure of FtsEX-EnvC complex from *E. coli*. Previously, they were unable to do so but were able to determine the structure of the complex from *P. aeruginosa*. Here they persisted in attacking the *E. coli* complex since more is known about its involvement in cell division and there is a wealth of mutants in *E. coli*. The structural work is well done and recapitulates the results this lab obtained with this complex from *P. aeruginosa*. It would be helpful to compare more directly the results obtained here with the *E. coli* complex with the previously reported *P. aeruginosa* complex - are they largely the same or has some insight been obtained from the work that was not present in the previous complex from *P. aeruginosa*. This is particularly the case in discussing the symmetrical FtsX dimer binding to the asymmetrical EnvC, since this is emphasized in the paper. However, Figures 3C & D of this paper appear similar to Figures 2D & E of the *P. aeruginosa* structure. Presumably, the additional information obtained and presented in Figure 4 is due to the higher resolution, but this needs to be highlighted and discussed to make it clear to a general audience.

The main issue is the model (Figure 6). In the model ATP is shown to bind to FtsEX before EnvC, however, in Figure 1c, it is shown that ADP is sufficient to promote binding of FtsEX to EnvC.

The work here is all done in vitro, however, information from in vivo needs to be considered. In vivo results reveal that the ATP-binding mutant FtsE(D162N)X promotes the recruitment of EnvC (Proc Natl Acad Sci U S A 2011 108:E1052-60). Thus, even FtsEX in vivo can bind EnvC without ATP (not sure if this mutant can bind ADP).

Perhaps the FtsE protein from *E. coli* has to have bound nucleotides to maintain its 3D structure.

Comments after revision:

The most interesting aspect of this complex is that it has yet to be determined the order of events in the ATPase cycle as the authors acknowledge. Although the authors have responded quite well to the comments, I am still worried about the significance of the in vitro results compared to the in vivo results reported by others. In vivo ATP binding does not appear required for complex formation (of course it is possible that ADP is responsible in vivo). Have the authors tried to solve the complex with ADP since they suggested that it is sufficient to hold the complex together. If possible, it would confirm the role of ATP binding by comparing the structures. Also, it is not clear if ADP binds to any of the mutants made by the Bernhardt lab (D162N, K41M). If they do not bind ADP then FtsEX without nucleotide is able to bind EnvC as the authors indicate is the case in Pseudomonas. It is also unclear the significance of the ATPase activity of FtsEX in vitro with or without EnvC. Could the activity be some basal activity that is not relevant to the in vivo situation. If EnvC caused FtsEX to hydrolyze ATP it would be a futile cycle as FtsEX and EnvC are localized to the septum long before they are involved septal hydrolysis.

# Referee report: Reviewer #2 (Public Review)

**DOI:** 10.7554/eLife.94336.3.sa2

Summary:

Peptidoglycan remodeling, particularly that carried out by enzymes known as amidases, is essential for the later stages of cell division including cell separation. In *E. coli*, amidases are generally activated by the periplasmic proteins EnvC (AmiA and AmiB) and NlpD (AmiC). The ABC family member, FtsEX, in turn, has been implicated as a modulator of amidase activity through interactions with EnvC. Specifically, how FtsEX regulates EnvC activity in the context of cell division remains unclear.

Strengths:

Li et al. make two primary contributions to the study of FtsEX. The first, the finding that ATP binding stabilizes FtsEX in vitro, enables the second, structural resolution of full-length FtsEX both alone (Figure 2) and in combination with EnvC (Figure 3). Leveraging these findings, the authors demonstrate that EnvC binding stimulates FtsEX-mediated ATP hydrolysis approximately two-fold. The authors present structural data suggesting EnvC binding leads to a conformational change in the complex. Biochemical reconstitution experiments (Figure 5) provide compelling support for this idea.

Weaknesses:

The potential impact of the study is curtailed by the lack of experiments testing the biochemical or physiological relevance of the model which is derived almost entirely from structural data.

Altogether the data support a model in which interaction with EnvC, results in a conformational change stimulating ATP hydrolysis by FtsEX and EnvC-mediated activation of the amidases, AmiA and AmiB. However, the study is limited in both approach and scope. The importance of interactions revealed in the structures to the function of FtsEX and its role in EnvC activation are not tested. Adding biochemical and/or in vivo experiments to fill in this gap would allow the authors to test the veracity of the model and increase the appeal of the study beyond the small number of researchers specifically interested in FtsEX.

Comments after revision:

Although I appreciate the authors' desire to save future biochemical experiments for a separate publication, the lack of in vitro data verifying their model makes it challenging to reconcile with published studies from other groups. The other reviewer's point about EnvC activating FtsEX ATPase activity resulting in a futile cycle since both are recruited to the septum well before constriction, is a good example of the disconnect between the model presented here and in vivo data.

# Author comment: Author response

**DOI:** 10.7554/eLife.94336.3.sa3

**Authors:** Jianwei Li, Yutong He, Xin Xu, Martin Alcorlo, Jian Shi, David I Roper, Juan A Hermoso, Lok-To Sham, Min Luo, Souvik Naskar, Nicholas S Briggs

**Affiliations:** National University of Singapore Singapore Singapore; National University of Singapore Singapore Singapore; National University of Singapore Singapore Singapore; Instituto Química Física Blas Cabrera Madrid Spain; National University of Singapore Singapore Singapore; University of Warwick Coventry United Kingdom; Instituto Química Física Blas Cabrera Madrid Spain; National University of Singapore Singapore Singapore; National University of Singapore Singapore Singapore; University of Warwick Coventry United Kingdom; University of Warwick Coventry United Kingdom

The following is the authors’ response to the original reviews.

**Reviewer #1 (Public Review):**

Summary:

In this paper, Li and colleagues overcome solubility problems to determine the structure of FtsEX bound to EnvC from *E. coli*.

Strengths:

The structural work is well done and the work is consistent with previous work on the structure of this complex from *P. aerugionsa*.

Weaknesses:

The model does not take into account all information that the authors obtained as well as known in vivo data.

The work lacks a clear comparison to the Pseudomonas structure highlighting new information that was obtained so that it is readily available to the reader.

The authors set out to obtain the structure of FtsEX-EnvC complex from *E. coli*. Previously, they were unable to do so but were able to determine the structure of the complex from *P. aeruginosa*. Here they persisted in attacking the *E. coli* complex since more is known about its involvement in cell division and there is a wealth of mutants in *E. coli*. The structural work is well done and recapitulates the results this lab obtained with this complex from *P. aeruginosa*. It would be helpful to compare more directly the results obtained here with the *E. coli* complex with the previously reported *P. aeruginosa* complex - are they largely the same or has some insight been obtained from the work that was not present in the previous complex from *P. aeruginosa*. This is particularly the case in discussing the symmetrical FtsX dimer binding to the asymmetrical EnvC, since this is emphasized in the paper. However, Figures 3C & D of this paper appear similar to Figures 2D & E of the *P. aeruginosa* structure. Presumably, the additional information obtained and presented in

Figure 4 is due to the higher resolution, but this needs to be highlighted and discussed to make it clear to a general audience.

The main issue is the model (Figure 6). In the model ATP is shown to bind to FtsEX before EnvC, however, in Figure 1c it is shown that ADP is sufficient to promote binding of FtsEX to EnvC.

The work here is all done in vitro, however, information from in vivo needs to be considered. In vivo results reveal that the ATP-binding mutant FtsE(D162N)X promotes the recruitment of EnvC (Proc Natl Acad Sci U S A 2011 108:E1052-60). Thus, even FtsEX in vivo can bind EnvC without ATP (not sure if this mutant can bind ADP).

Perhaps the FtsE protein from *E. coli* has to have bound nucleotides to maintain its 3D structure.

Thank you for your thoughtful feedback and valuable suggestions. We have carefully revised the manuscript to address these concerns, incorporating additional analysis and discussion to enhance clarity and improve the accuracy of our interpretation.

Regarding the relationship between EnvC binding and nucleotide binding to FtsEX, our previous study on *P. aeruginosa* FtsEX demonstrated that FtsEX can bind EnvC even in the absence of nucleotide (PMID: 37186861, Fig. 3C). However, for *E. coli* FtsEX (Fig. S1 in this study), ATP is required to stabilize the complex in vitro, preventing us from directly testing whether EnvC binding is ATP-dependent. The reviewer raised an important point about the FtsED162N mutant study, from which previous studies suggests that this mutant may still retain ATP binding, as observed in its homolog MacB (PMID: 29109272, PMID: 32636250). Additionally, previous work (PMID: 22006325) has shown that the PLD domain of FtsX can bind EnvC directly, even in the absence of the NBD domain, a finding further supported by Crow’s lab (PMID: 33097670). Taken together, these studies indicate that EnvC binding to FtsEX is likely nucleotideindependent, while ATP binding primarily stabilizes FtsE dimerization, reinforcing FtsEX complex formation.

In line with these findings, our results suggest a stabilizing role of ATP in FtsEX assembly, whereas EnvC binding does not appear to be nucleotide-dependent. However, we acknowledge that the precise sequence of ATP binding and EnvC recruitment within the cell remains unresolved. To reflect this, we have revised the manuscript to incorporate these insights (L190-201, L445-451), clearly stated the limitations (L450-451, L887-890), and updated our model (Fig. 6) to avoid assigning a definitive sequence to EnvC and ATP binding.

Additionally, we have strengthened the structural comparison between *E. coli* and *P. aeruginosa* FtsEX, as the reviewer suggested. We have now included a detailed comparative analysis (L282-306, Fig. S9), which reveals that the transmembrane and nucleotide-binding domains are highly superimposable. The primary structural distinction lies in a slight tilting difference in the bound EnvC, which appears to stem from the conformation of the X-lobes within the PLD domains. Highlighting these differences helps clarify how our new structural data provide additional insights beyond what was previously observed in *P. aeruginosa*.

**Reviewer #2 (Public Review):**

Summary:

Peptidoglycan remodeling, particularly that carried out by enzymes known as amidases, is essential for the later stages of cell division including cell separation. In *E. coli*, amidases are generally activated by the periplasmic proteins EnvC (AmiA and AmiB) and NlpD (AmiC). The ABC family member, FtsEX, in turn, has been implicated as a modulator of amidase activity through interactions with EnvC. Specifically how FtsEX regulates EnvC activity in the context of cell division remains unclear.

Strengths:

Li et al. make two primary contributions to the study of FtsEX. The first, the finding that ATP binding stabilizes FtsEX in vitro, enables the second, structural resolution of fulllength FtsEX both alone (Figure 2) and in combination with EnvC (Figure 3). Leveraging these findings, the authors demonstrate that EnvC binding stimulates FtsEX-mediated ATP hydrolysis approximately two-fold. The authors present structural data suggesting EnvC binding leads to a conformational change in the complex. Biochemical reconstitution experiments (Figure 5) provide compelling support for this idea.

Weaknesses:

The potential impact of the study is curtailed by the lack of experiments testing the biochemical or physiological relevance of the model which is derived almost entirely from structural data.

Altogether the data support a model in which interaction with EnvC, results in a conformational change stimulating ATP hydrolysis by FtsEX and EnvC-mediated activation of the amidases, AmiA and AmiB. However, the study is limited in both approach and scope. The importance of interactions revealed in the structures to the function of FtsEX and its role in EnvC activation are not tested. Adding biochemical and/or in vivo experiments to fill in this gap would allow the authors to test the veracity of the model and increase the appeal of the study beyond the small number of researchers specifically interested in FtsEX.

Thank you for your thoughtful review and constructive feedback. We appreciate your recognition of our study’s contributions, particularly the structural resolution of fulllength *E coli* FtsEX, its interaction with EnvC, and our biochemical characterization of EnvC-stimulated ATP hydrolysis.

We understand the importance of further biochemical and in vivo validation to support our model. While our study primarily provides a structural framework for understanding FtsEX function, many key residues identified in our *E. coli* structures have already been tested in prior cell physiological studies. For example, residues critical for the FtsEXEnvC interaction were examined in our collaborator David Roper’s lab in collaboration with Crow’s lab (PMID: 33097670, L319-321).

With the structural blueprint provided by our full-length *E. coli* FtsEX-EnvC complex, we now have a foundation to explore several key functional aspects of this system. Future mutagenesis studies will help dissect the roles of specific residues in ATP binding/hydrolysis, coupling between the TMD and NBD domains, interactions between the PLD and TMD domains of FtsX, and signal transduction from the NBD, through the TMD and PLD to EnvC. Additionally, we aim to investigate how the symmetrical PLD domain recruits asymmetrical EnvC and how the dynamics of PLD of FtsX and CCD domains of EnvC contribute to the complex’s function.

As these experiments require specialized expertise in cell physiology and PG degradation assays, we are actively collaborating with experts in these areas to pursue them. We are committed to furthering this work and providing deeper biochemical and in vivo insights into the function of the FtsEX complex in cell division.

**Reviewer #1 (Recommendations For The Authors):**

(1) As mentioned, two things could strengthen the paper. One is to take into account that ADP or possibly nucleotide-free FtsEX can bind EnvC. The second is to highlight any differences between the structures from *E. coli* and *P. aeruginosa*.

Thank you for these insightful suggestions. In our revision, we have (1) carefully considered the possibility of EnvC binding independently of nucleotide and (2) have incorporated a detailed comparison between the newly obtained *E. coli* FtsEX/EnvC structure and that of *P. aeruginosa*.

Regarding the relationship between EnvC binding and ATP binding to FtsEX, our previous study on *P. aeruginosa* FtsEX demonstrated that FtsEX can bind EnvC in the absence of nucleotide (PMID: 37186861, Fig 3C). However, for *E. coli* FtsEX systems (Fig S1 in this study), ATP is necessary for FtsEX stabilization in vitro, which limited us from further directly testing whether EnvC binding is ATP-dependent or not.

We appreciate the reviewer’s reference to the FtsE(D162N) mutant study. Previous studies suggest that D162N mutant may still retain ATP binding, similar to its homolog MacB (PMID: 29109272; PMID: 32636250). Additionally, findings from Winkler’s lab (PMID: 22006325) indicate that the PLD domain of FtsX can bind EnvC directly, even in the absence of the NBD domain, a result further supported by study from Crow’s lab (PMID: 33097670). Collectively, these studies suggest that EnvC binding to FtsEX is nucleotide-independent, while ATP binding likely stabilizes FtsE dimerization, thereby reinforcing FtsEX complex formation, as the reviewer suggested.

Thus, consistent with previous studies, our results so far support a stabilizing role of ATP in FtsEX assembly, while EnvC binding itself does not appear to be nucleotidedependent. However, the available evidence remains inconclusive, and the precise sequence of ATP binding and EnvC recruitment within the cell is still unclear. In our revision, we have now incorporated these analyses in L190-201 and L445-451, stated the limitations (L450-451 and L887-890) and updated our model (Fig. 6) to avoid assigning a definitive sequence to EnvC and ATP binding.

For the structural comparison between *E. coli* and *P. aeruginosa* FtsEX, we have added a detailed analysis in L282-306 and Supplementary figure 9. In summary, we found that the transmembrane domain and nucleotide-binding domain are highly superimposable, with only minor differences observed. The primary distinction lies in a slight tilting difference in the bound EnvC, which appears to come from the conformation of the X-lobes within the PLD domains.

(2) Line 129. Concerning the role of ATP in stabilizing the complex. It is clear that ADP can do it as well (Figure 1c). This is mentioned in line 131 but not considered in the model.

Thank you for pointing this out. We have now revised the relevant sections in the manuscript (L190-201 and L445-451) and updated the model (Fig 6) accordingly. In the revised manuscript, we acknowledge the reviewer’s point that ATP may primarily serve to stabilize the FtsEX complex. Additionally, we have explicitly clarified that EnvC binding appears to be nucleotide-independent. Regarding the model, we state that the current study does not provide sufficient evidence to determine the precise sequence of EnvC and ATP binding to FtsEX in the cell. We believe these revisions, incorporating the reviewer’s suggestions, improve the accuracy of our interpretation.

**Reviewer #2 (Recommendations For The Authors):**

(1) The introduction is written for an audience with significant expertise in bacterial PG synthesis and is thus difficult for those outside the field to follow.

Thank you for your feedback. We have revised the introduction, particularly the first passage (L51–63), to improve readability and make it more accessible to a broader audience.

(1) Figure 1: Please express ATP hydrolysis data in ATP/FtsEX/minute. (It is currently nmol/mg/min).

Changed accordingly, thank you!

(2) Figure 4: Please clarify in the legend and in the figure itself which structures correspond to full-length data from cryoEM data or truncated (FtsEX-PLD domain) protein data from previous crystallographic studies.

Both the FtsEX and FtsEX/EnvC complex structures shown in Figure 4 were obtained from our cryo-EM data using full-length proteins. To avoid any confusion, we have now further clarified this in the figure legend (L857).
